# Assessment of myocardial oxygenation changes in the presence of coronary artery stenosis with three dimensional cardiac phase-resolved SSFP BOLD imaging in canines

**DOI:** 10.1186/1532-429X-11-S1-P32

**Published:** 2009-01-28

**Authors:** Xiangzhi Zhou, Sven Zuehlsdorff, Saurabh Shah, Richard Tang, Rachel Klein, Debiao Li, Rohan Dharmakumar

**Affiliations:** 1 Department of Radiology, Northwestern University, Chicago, IL USA; 2 Siemens MED US, Chicago, IL USA

**Keywords:** Coronary Artery Stenosis, Adenosine Stress, Oxygen Sensitivity, Regional Signal Difference, Left Circumflex Coronary Artery

## Introduction

Recent studies have shown that regional myocardial oxygenation changes secondary to coronary artery stenosis may be detected with 2D steady-state free precession(SSFP) blood-oxygen level-dependent(BOLD) imaging. One potential limitation of the 2D SSFP BOLD approach is the disruption of steady-state myocardial signal due to through-plane cardiac motion in the presence of slice-selective excitation. A consequence of breaking the dynamic steady state of SSFP BOLD signal is that it may lead to a reduction in myocardial oxygen sensitivity.

## Purpose

To investigate whether there are any observable differences in myocardial BOLD contrast between 2D and 3D approaches using a canine model with adjustable coronary artery stenosis

## Methods

Four dogs were operated and studied under institutional approval. For each dog, following thoracotomy, a hydraulic occluder was placed around the left circumflex coronary artery (LCX) to induce reversible LCX stenoses. A Doppler flow probe was placed distal to the LCX occluder to assess the fidelity of the occlusion during the MRI studies. Following recovery (1 week), animals were sedated, ventilated and placed on the scanner table (1.5 T Siemens Espree). ECG-gated and multiple breath-held 2D and 3D cine SSFP sequences were prescribed under basal and adenosine stress with and without LCX stenosis over the LV. Typically 2–3 stenosis levels (on the basis of Doppler flow) were assessed and each animal was studied 2–3 times. Scan parameters for 2D acquisitions: in-plane resolution = 1.2 × 1.2 mm^2^, slice thickness = 5 mm, iPAT factor = 2, TR/TE = 4.7/2.35 ms, flip Angle = 70°; 3D acquisitions were the same, except for slab thickness = 30 mm(6 partitions). Both 2D(3 slices, 5 mm gap) and 3D short-axis images were acquired with center slice positioned over mid left-ventricle. TR and segments/cardiac phase were adjusted to achieve the optimal temporal resolution (10 ms to 20 ms) and minimize motion and flow artifacts in 2D and 3D acquisitions, while maximizing oxygen sensitivity.

### Data analysis

Regional SSFP BOLD Contrast, defined as [I_LAD_-I_LCX_]/I_LAD_, where I_LAD_ and I_LCX_ are average SSFP signal intensity of region LAD and LCX, respectively, was used to evaluate the SSFP BOLD images at end diastole. Contrast values computed from 2D and 3D acquisitions were averaged separately for each study under basal and stenotic conditions. Two sample t-tests were performed to test whether (1) there were regional signal differences between basal and stenotic conditions for 3D BOLD SSFP and (2) to test where there were differences in regional SSFP BOLD Contrast between 2D and 3D acquisitions under stenotic conditions. Statistical significance was set at p < 0.01.

## Results

**Figure 1:**
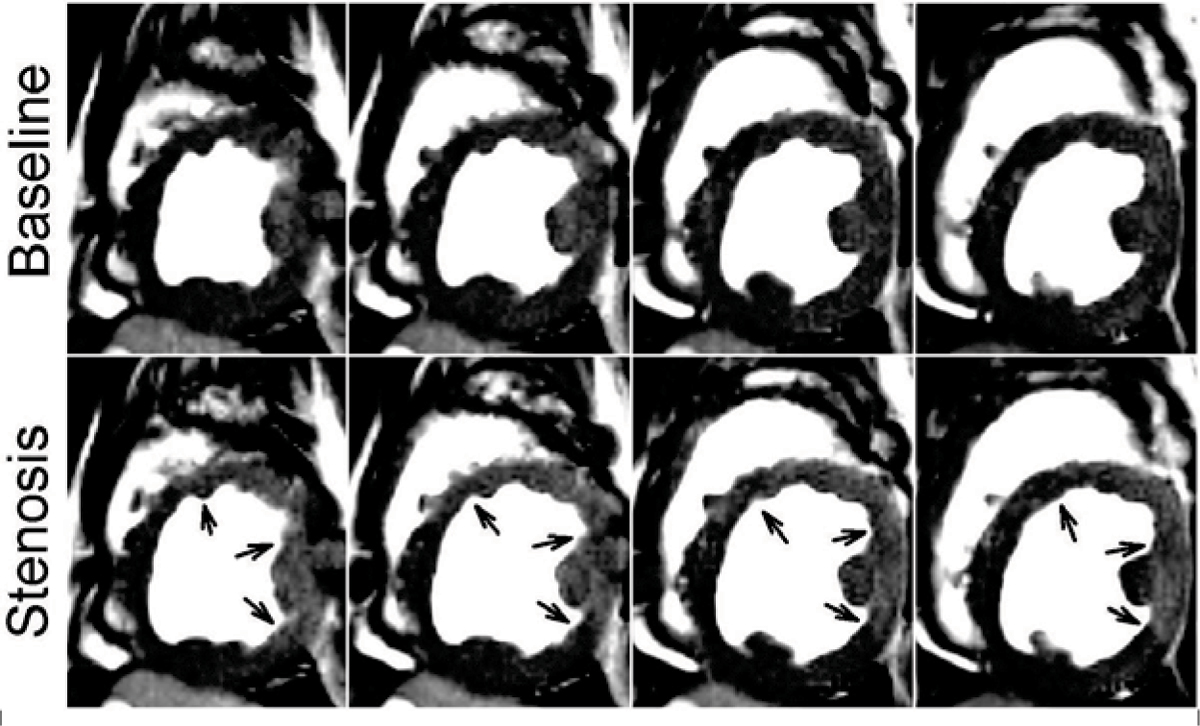
**A representative set of end-diastolic 3D SSFP BOLD images (4 contiguous slices from apex to base (obtained from a dog under baseline condition (upper row) and with moderate stenosis (lower row) under adenomise stress is shown**. Note the signal enhancement observed in the non-LCX supplying territories (arrows) uder stress, while no signal enhancement is observed within the LCX supplying region in the presence of adenosine.

**Figure 2:**
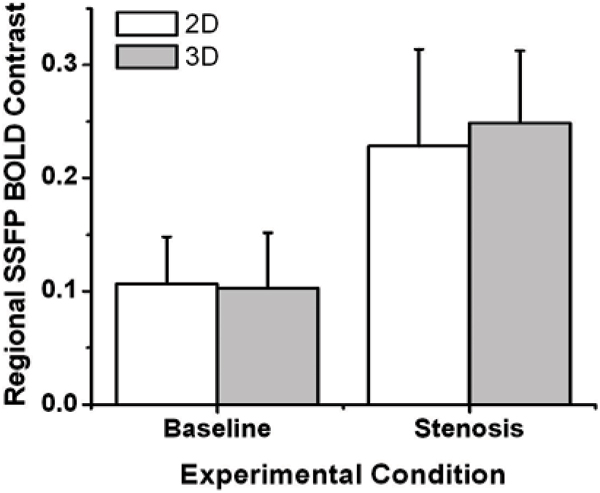
**Regional 2D and 3D SSFP BOLD signal contrast between LAD and LCX regions under baseline conditions and steosis (with adenosine)**. There were no significant differences in regional signal intensity variations at baseline and under stress (with stenosis) between 2D and 3D images. However, statistically significant differences were found for regional SSFP BOLD contrast between LAD and LCX territories in the presence of stenosis compated to baseline conditions with both 2D and 3D approaches. (P < 0.01).

Figure 1 shows a representative set of end diastolic 3D SSFP BOLD images (4 contiguous slices from apex to base) obtained from a dog under basal condition (upper row) and with moderate stenosis under adenosine stress (lower row). Note that under adenosine stress, the signal intensities within myocardial territories not supplied by the LCX enhance while that supplied by LCX remain isointense with the rest images. Results from quantitative analysis are shown in Figure 2. No statistically significant regional signal differences were found between 2D and 3D acquisition under baseline or LCX stenosis under adenosine stress (p > 0.2). There were statistically significant differences between baseline and LCX stenosis under adenosine stress for both 2D and 3D acquisitions.

## Conclusion

While 2D SSFP BOLD have shown promising results, reduction in oxygen sensitivity due to through-plane motion was a notable potential limitation. Results from this study showed that oxygen sensitivity between 2D and 3D acquisitions are not significantly different, demonstrating that through-plane motion does not significantly alter myocardial SSFP BOLD contrast. Further extension of 3D cine BOLD imaging with improved artifact reduction permitting the use of higher TR for improved oxygen sensitivity, faster data acquisition strategies, and free-breathing approaches may be necessary prior to successful clinical translation of 3D SSFP BOLD MRI.

